# Multi-Objective Multi-Instance Learning: A New Approach to Machine Learning for eSports

**DOI:** 10.3390/e25010028

**Published:** 2022-12-23

**Authors:** Kokten Ulas Birant, Derya Birant

**Affiliations:** Department of Computer Engineering, Dokuz Eylul University, Izmir 35390, Turkey

**Keywords:** machine learning, multi-instance learning, classification, eSports

## Abstract

The aim of this study is to develop a new approach to be able to correctly predict the outcome of electronic sports (eSports) matches using machine learning methods. Previous research has emphasized player-centric prediction and has used standard (single-instance) classification techniques. However, a team-centric classification is required since team cooperation is essential in completing game missions and achieving final success. To bridge this gap, in this study, we propose a new approach, called *Multi-Objective Multi-Instance Learning* (MOMIL). It is the first study that applies the multi-instance learning technique to make win predictions in eSports. The proposed approach jointly considers the objectives of the players in a team to capture relationships between players during the classification. In this study, *entropy* was used as a measure to determine the impurity (uncertainty) of the training dataset when building decision trees for classification. The experiments that were carried out on a publicly available eSports dataset show that the proposed multi-objective multi-instance classification approach outperforms the standard classification approach in terms of accuracy. Unlike the previous studies, we built the models on season-based data. Our approach is up to 95% accurate for win prediction in eSports. Our method achieved higher performance than the state-of-the-art methods tested on the same dataset.

## 1. Introduction

*Electronic sports* (eSports) is a general term used to describe online digital games that are played professionally or amateurishly by teams and watched by a large number of audiences. Naturally, eSports is a significant research area in both the scientific community and industry, in terms of not just size, but also commercial value [1]. eSports games generate huge amounts of statistical match data that are publicly available, allowing us to extract significant insights. Multiplayer Online Battle Arena (MOBA) provides a great opportunity for machine learning (ML) with the availability of high-volume and high-dimensional data. The data dimension is high since it involves many different attributes in three main categories (pre-game features, in-game features, and post-game features) such as player-related information (e.g., champion character, role, and position), match-related information (e.g., season, platform, duration, and software version), team-related information (i.e., kills, assists, deaths, damages, healing, wards, vision score, level, rewards, bans, and golds earned/spent for each player separately), information regarding coaches and trainers, characters to be selected, items to be purchased, and statistics (e.g., blood, tower, inhibitor, dragon, baron, and win–lose). Formally, standard *eSports analytics* was defined by Schubert et al. [2] as follows: the process of using eSports-related data to find and visualize useful and meaningful patterns/trends to assist with decision-making processes. This definition highlights a fundamental aspect: the opportunity of using machine learning techniques to predict match outcomes.

The eSports industry has already become a highly profitable industry with total revenue of $159.3 billion [3] and over 645 million audiences [4]. The prediction of eSports competition has important impacts on market size and growth, revenue, sponsorship, and media coverage [5]. Therefore, forecasting match outcomes is requested by all the stakeholders such as professional players, amateurs, coaches, trainers, organizers, sponsors, audiences (fans), and media workers. In this way, they can develop tactics to gain advantages in eSports competitions [6,7]. To meet this demand, ML models have been developed in several studies [8,9,10]. In other words, the strengths of ML methods in making predictions related to eSports have been proven in previous studies [11,12].

The previous studies [6,7,8,9,10,11,12,13,14,15] related to prediction in eSports have several limitations. First, some studies [8,9] have been mainly focused on in-game predictions, aiming to inform audiences and players. Second, some of them [16,17] have built models for player skill prediction, and so they used sensors such as eye trackers, keyboard/mouse loggers, electroencephalography (EEG) headsets, pulse-oximeters, heart rate monitors, and chair seat/back sensors, which limit the area of its application due to hardware requirements. Third, they [9,10,18] used data collected over long time periods; however, eSports games are continuously updated, and these major game changes can remarkably alter the fundamental characteristics of the games. Since major updates might make previous data obsolete, we carried out a season-based analysis in order to overcome this limitation. Last and foremost, the previous studies [8,15,16,17,19] are player-centric and use a standard (single-instance) classification technique. However, the strong performance of a single player does not guarantee a win for the team, and a weak performance of a single player does not guarantee a loss [7]. Team-level classification is required since team cooperation is essential in completing game missions and achieving final success. To be able to predict the match outcome by considering multiple players in a team, multiple instance classification is required; however, no previous prediction models have been adopted for multi-instance learning.

*Multi-instance learning* (MIL) [20] is a special type of machine learning (ML) where multiple training samples are assigned into bags and only one class label is assigned for all the samples in a bag. In other words, it learns from a set of training bags that involves multiple feature vectors. In our study, each bag corresponds to a team and contains five separate feature vectors for five players in the team. If the team wins the game, its bag is associated with the class label 1, and otherwise, the label is 0. We propose using MIL since collaboration among players on each team remarkably affects which team will win or lose.

The novelty and contributions of this article can be summarized as follows. (i) It proposes a new approach, called *Multi-Objective Multi-Instance Learning* (MOMIL). (ii) It is the first study that applies the *multi-instance learning* technique to predict the match outcome in eSports. (iii) It considers the multi-objective concept in MIL for the first time, since each team member has a specific role (objective) in eSports matches. Importantly, the aim of our study is to provide an algorithmic contribution towards increasing the classification performance of models compared to the previous studies [13,21,22,23,24]. (iv) Unlike previous studies, we built the models on season-based data due to the regularity of changes and updates in the game versions. Thereby, our study is also original in that it considers model usage lifespan first-time and presents extended results and analyses for win prediction. (v) Our method achieved higher performance than the state-of-the-art methods [13,21,22,23,24] tested on the same dataset.

*Entropy* is the elementary measure used in this study when building decision trees. The experiments that were carried out on a publicly available LoL dataset show that the proposed approach outperforms the standard classification approach in terms of accuracy. Using multi-instance learning algorithms, our approach is up to 95% accurate for win prediction in LoL seasons.

The remainder of the article is organized as follows. In the following section, we provide a comprehensive literature review of ML in eSports. Furthermore, we give background information on LoL and provide definitions of multi-objective and multi-instance learning. Section 3 explains the proposed MOMIL approach. Section 4 describes four different experiments and the experimental results. The Section 5 summarizes the study and suggests possible future works.

## 2. Related Work

### 2.1. Literature Review

eSports is currently referred to as one of the major international and popular sports with millions of amateur and professional players and spectators. Unlike original sports, eSports can be performed without being dependent on place and time in its nature; therefore, a large number of matches have been held every day, resulting in a huge amount of data and opportunities for ML studies.

Table 1 shows a comparison of our study with the previous studies [6,7,8,9,10,11,12,13,14,15,16,17,18,19,23,24,25,26,27,28,29,30,31,32]. It provides a brief description, an overview of algorithms, the task performed, the genre of the game, and whether multi-instance learning was adopted or not. Many studies have been focused on *classification* (CF) [6,7,8,9,10,12,13,15,16,17,19,23,24,28,31] and *regression* (R) [11,14,18,27,32] tasks; however, recently, *clustering* (CL) [29,30], and *association rule mining* (ARM) [24] tasks have received increasing attention from researchers.

eSports analytics research has focused on different problems such as predicting match outcomes [6,7,10,12,13,14,15], recommending items [23,24], predicting the ranking of players [18], classifying eSports games [28], identifying roles [29], predicting player churn [32], clustering player-centric networks [30], predicting player skill, and re-identification [16,17]. Since win prediction is one of the most important problems and it has commercial value, in this study, we focused on this problem. However, match outcome prediction in eSports is very different from the win prediction in physical sports [33,34].

In the literature, a variety of ML algorithms have been used for prediction problems in eSports. Much of the previous work [6,7,8,9,10,16,23,24,27] used Linear or Logistic Regression (LR); however, other regression methods such as Mixed-Effects Cox Regression (MECR) [32] and Bayesian Hierarchical Regression (BHR) [14] have also been applied to solve a prediction problem in eSports. The main drawback of linear regression is that it can model linear dependencies in the data. On the other hand, the disadvantage of logistic regression is the difficulty of detecting complex relationships between data instances. The most widely used classification techniques are Neural Networks (NN) [8,11,12,23,24], Decision Tree (DT) [23,24,27,28,31], Classification and Regression Tree (CART) [7,12], K-Nearest Neighbors (KNN) [8,13,15,16,31], Support Vector Machine (SVM) [15,16,27], and Naive Bayes (NB) [16,28]. However, building a single classifier with these techniques may not be strong and stable enough. This issue can be overcome by using an ensemble model. As types of ensemble learning, the Random Forest (RF) [6,10,16,18,28,31], Gradient Boosting Machines (GBM) [6,10,18], and Extremely Randomized Trees (ERT) [17] methods were tested in some studies. The advantage of ensemble learning over the single classifier is the ability to combine the prediction outputs from multiple estimators to improve generalization ability and robustness. An incorrect prediction of an ensemble member can be corrected by other members thanks to the majority voting. However, one of the drawbacks of ensemble learning techniques is that they increase computation time and model complexity. The sequential nature of team fights in eSports may make non-deep learning models less suitable. Therefore, deep learning techniques such as Long Short-Term Memory (LTSM) [25], Deep Neural Network (DNN) [19], and Convolutional Neural Networks (CNN) [12,23,26] have also been used in predicting match outcomes. Principal Component Analysis (PCA) [31], Linear Discriminant Analysis (LDA) [15], and Quadratic Discriminant Analysis (QDA) [15] methods have also been applied to eSports data. The drawback of PCA and LDA techniques is that they define a linear projection of the data, and therefore, the scope of their application is somewhat limited. On the other hand, QDA has the advantage of separating non-linear data.

Multi-instance learning [35] is one of the most exciting technologies that implement entropy in computer science [36]. The entropy of a dataset is a good measure to determine its impurity or unpredictability. In this study, we used entropy to characterize the structure of the data when building a decision tree. In addition to multi-instance models, multi-objective [37] and multi-strategy models [38] have also been successfully developed in the literature.

Some studies in the literature have mainly used pre-game features [10] or in-game features [6], while we consider post-game features. *Pre-game features* are generated in the character and item selection phase before a match starts. For example, Araujo et al [23,24] recommend giving the most suitable item set to each team member to increase the performances of their characters. *In-game features* are used for live (real-time) predictions. For instance, Katona et al. [19] predicted whether a player will die within the next five seconds. *Post-game features* are generated to summarize the game, notably at the end of the game, such as kills, rewards, damages, gold earned by each player, and match duration. Kadan et al. [31] subdivided games into several intervals and predicted the round results.

The majority of work in eSports has focused on constructing ML models for well-known games, including League of Legends (LoL) [8,9,11,12,13,16,23,24,28,30,32], Defense of the Ancients 2 (Dota 2) [6,7,10,14,19,28,29], PlayerUnknown’s Battlegrounds (PUBG) [18,27], Counter-Strike (CS) [17,28,31], and StarCraft [15]. Since LoL is the most popular game in eSports in the world, in this study, we focused on this game.

Our study differs from existing studies in many respects. First, our study is the first study that uses multi-instance learning in eSports. Second, data analysis techniques in the previous studies are mainly classical ML algorithms such as LR, SVM, RF, DR, NN, and KNN, while we used different algorithms such as Multi-Instance Tree Inducer (MITI), Multi-Instance Rule Inducer (MIRI), Two-Level-Classification (TLC), and Multi-Instance Wrapper (MIWrapper) algorithms. Third, it is designed to be season-based since each season involves many changes in the meta-game that affect winner prediction. In this way, we overcome the limitations of current studies, meaning our approach generalizes well to different game versions.

### 2.2. Background Information

#### 2.2.1. League of Legends

LoL is a MOBA game, where the blue team and the red team compete against each other on a single map (called *Summoner’s Rift*) to destroy the opposing base (called *Nexus*) first. The map contains three main roads (called *lanes*), which are connected by a base of each team. The game typically consists of five players on each team, and each player (called *summoner*) controls a single character (called *champion*). Once the match begins, each player selects a champion to play over 120 champions and goes to their respective lane after buying some starting items with golds. *Items* are objects (e.g., weapons, armor) that are used for providing an improvement on champions. *Gold* or *experience points* (XP) is the in-game currency for an LoL match to buy performance-enhancing items. There are also computer-controlled monsters such as Dragon, Baron, and Rift Herald. Throughout a match, the players gain gold and experience in a variety of ways such as killing enemy champions or monsters and destroying defense buildings (called *Towers*) of the enemy team. When a player is killed, their champion is reanimated after a time-out increasing in accordance with the champion’s XP. Each match lasts approximately 20–40 min on average. Finally, whichever team destroys the Nexus in the opponent’s base first obtains the victory. Players are ranked according to their skill levels (called *ladder*) as follows: Bronze, Silver, Gold, Platinum, Diamond, Master, and Challenger.

LoL is a team game and the final performance in the game under investigation depends on the relationship among players in the team [7]. At the beginning of the game, each player in a five-member team selects a single character, which has various advantages and disadvantages to contribute to the overall strategy of the team [39]. The ability of a character can become more valuable with its harmony with other characters. In other words, the ability of a character can be dependent on the interaction with another characters. As it is a team-based game, each player in the team typically plays a certain role (objective) in a match, similar to traditional sports. For example, in the LoL game, team members play in a cooperative manner by selecting a position, including top lane, mid lane, jungle, attack damage carry, and support. Players in a team influence each other by means of cooperation; e.g., one player can defend another player who is attacking the enemy. The studies [7,39,40] in the literature have emphasized the importance of teamwork and compromise. For example, Lan et al. [39] indicated that the outcome of a game is determined both by each player’s behavior and by the interactions among players. Gu et al. [40] also noted that cooperation among teammates should be considered for match outcome prediction. As emphasized by a number of studies [7,39,40], the objectives, positions, and abilities of five players are all together relevant for the team’s success. Since team collaboration is critical for success, we propose a team-level classification approach in this paper.

#### 2.2.2. Multi-Objective and Multi-Instance Learning

*Multi-objective learning* is a type of learning in which each member has a special role (task) and has a probability of reaching the target goal, and the final result is determined by jointly considering the features of members. For example, in an eSports match, each player is responsible for one task on a team and has a success rate, players influence each other by means of cooperation, and the team with the better performance wins.

*Multi-instance learning* (MIL) is a special case of learning where the algorithm learns from bags of instances, rather than single instances. The aim of MIL is to classify bags according to several unseen instances.

**Figure 1:**
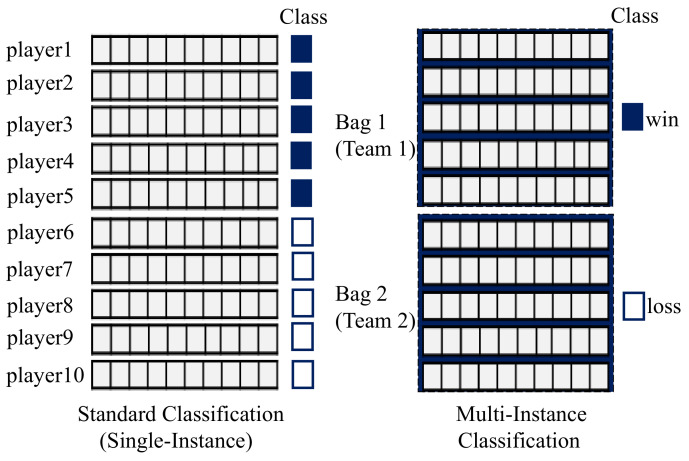
Standard classification versus multi-instance classification.

Figure 1 illustrates the difference between the standard classification and multi-instance classification. In traditional classification, each instance (a feature vector) is assigned to a certain class label. Nevertheless, in multi-instance classification, a training set consists of bags containing several feature vectors, and each bag is associated with a class label. In other words, a multi-instance classification algorithm learns from a dataset that contains bags of training instances, rather than single training instances. In our study, each bag corresponds to a team in the LoL game and contains five separate feature vectors (match statistics) for five players belonging to the team. If a team wins the game, its bag is associated with the class label 1, and otherwise the label is 0.

## 3. Materials and Methods

### 3.1. The Proposed Approach

Using the standard (single-instance) classification to estimate the match outcome of an eSports game has attracted considerable attention [6,7,10,12,13,14,15]; however, multi-instance classification is as yet unknown. Multi-instance classification is, however, required because it provides the ability to jointly consider multiple instances (here multiple players in a team) when classifying. In order to bridge this gap, this paper proposes a new approach: MOMIL.

MOMIL learns from multi-instance data, builds a classification model by simultaneously considering multiple instances, and then uses the model to predict the winning probability of a team for unseen vectors. Considering data from multiple players jointly can help to capture relationships among players and to explore team-level mechanics that are particularly relevant since most of the games in eSports are team-based games. Our study especially investigates the winning predictors in LoL.

**Figure 2:**
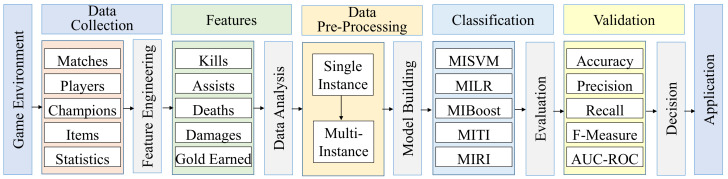
The general overview of the proposed MOMIL approach.

Figure 2 presents the general overview of the proposed MOMIL approach, which consists of five main stages. (i) In the first stage, named *data collection*, the raw data are collected from the game environment, including information about matches, champions, players, items purchased, and statistics. (ii) In the second stage, *features* are generated, which summarize the games, including kills, assists, deaths, damages, rewards, and golds earned by each player. (iii) In the *data pre-processing* stage, single-instance data are transformed into multi-instance data. Since each team in LoL is composed of five players, every five sequential records in the training set were merged in a straightforward manner, from solo to full team composition. (iv) In the next step, predictive models were built by using multi-instance learning algorithms such as Multi-Instance Support Vector Machines (MISVM), Multiple Instance Logistic Regression (MILR), Multi-Instance AdaBoost (MIBoost), Multi-Instance Tree Inducer (MITI), and Multi-Instance Rule Inducer (MIRI). (v) In the fifth stage, the predictive models are tested to evaluate their performances by using various metrics such as accuracy, recall, precision, F-measure, and the area under the curve of the receiver operating characteristic (AUC-ROC). In this stage, the k-fold cross-validation technique is used for validation, in which the data are divided into *k* equal subsets, using k−1 folds as the training set and one fold as the test set. Finally, in the win prediction stage, the match outcome is predicted according to a given sample.

The aim of this study is to develop an intelligent model that discovers useful patterns and rules to be able to estimate the match outcomes of eSports games. Our approach (MOMIL) improves win prediction as it makes team-level analysis by taking into account the objectives of the players in the team together during the classification. It investigates the correlation between match outcome (class label) and multiple objectives simultaneously rather than individually.

The comparison of our approach against traditional ones can be summarized as follows. Rather than player-centric prediction, we performed a team-centric prediction. Instead of using standard single-instance classification algorithms, we used multi-instance learning algorithms. Our approach considers the multi-objective concept since each team member has a specific role (objective) in eSports matches. Unlike making predictions over long time periods, we built the models on season-based data since each season involves many changes in the environment.

### 3.2. Formal Description of the Proposed Approach

Let *X* is a *d*-dimensional instance such that $X=(x_{1},x_{2},\ldots ,x_{d})$. Let *O* objective types such that $O=\{O_{1},O_{2},\ldots ,O_{m}\}$. A bag *B* includes a set of pairs of instances and their obejectives such that $B=(\left(X_{1},O_{1}\right),\left(X_{2},O_{2}\right),\ldots ,\left(X_{m},O_{m}\right))$, where *m* is the number of intances in a bag, as well as the count of objectives. Training dataset *D* contains a set of pairs of bags and their corresponding class labels such that $D=\{<B_{1},C_{1}>,<B_{2},C_{2}>,\ldots ,<B_{n},C_{n}>\}$, where $C_{i}$ is the class label of the bag $B_{i}$. The target class attribute has *k* labels such that $C_{i}\in \left\{C_{1},C_{2},\ldots .,C_{k}\right\}$ for i=1,2,…,k. For instance, in a binary classification, the class labels of the bags can be one or zero, i.e., $C_{1}=1$ and $C_{2}=0$. The aim is to build a multi-objective multi-instance classifier model *M* to successfully label given query bags. The multi-instance classifier predicts the output at the bag-level and makes a decision for a given query bag. In fact, a traditional classifier is a special type in which each bag includes only one instance, as well as only one objective $B_{i}=\left(X_{i}\right)$.

**Definition 1**. *MOMIL is an approach that is applied to a dataset that consists of a set of bags $D={\left\{<B_{i},C_{i}>\right\}}_{i=1}^{n}$, wgere each bag contains a set of instances, each of which has an objective such that $B=(\left(X_{1},O_{1}\right),\left(X_{2},O_{2}\right),\ldots ,\left(X_{m},O_{m}\right))$. First, a multi-objective multi-instance dataset (D) is generated from a traditional (single-instance) training set (S), and then a classifier model (M) is trained directly on such data. In this way, the MOMIL approach jointly considers relationships and cooperation among data instances during classification.*

Algorithm 1 presents the pseudo-code of the proposed MOMIL method. In the first loop, the single-instance data (*S*) are transformed into multi-instance data (*D*). When each team in the game is composed of *m* players, every sequential *m* records in the data are merged in a straightforward manner, from solo to full team composition. In this way, the player-level match statistics are taken into account as single-instance data, while team-level features are considered as multi-instance data. After that, the algorithm builds the model (*M*) on multi-instance data. The constructed model maps input vectors representing game statistics of the players in the teams to output labels (win or loss). Finally, in the last loop, the winners are estimated for the given test data. At the *i*th iteration, the test query $T_{i}$ is classified by the model *M* and the predicted class labels are stored in a data structure (*Y*). The time complexity of the proposed MOMIL approach is O(L(n)+n∗m), where *m* is the number of players in a team, *n* is the number of bags in the training set, and *L* is the time required for the learning process on *n* bags.
**Algorithm 1:** Multi-Objective Multi-Instance Learning (MOMIL)**Inputs:** *S*: single-instace data $S=\{\left(x_{1},y_{1}\right),\left(x_{2},y_{2}\right),\ldots .,\left(x_{n*m},y_{n*m}\right)\}$ *T*: test set containing *b* bags *m*: the number of players in a team *n*: the number of bags in the training set**Outputs:** *Y*: predicted class labels on given query bags**Begin:****for:**i=1***to****n***do**
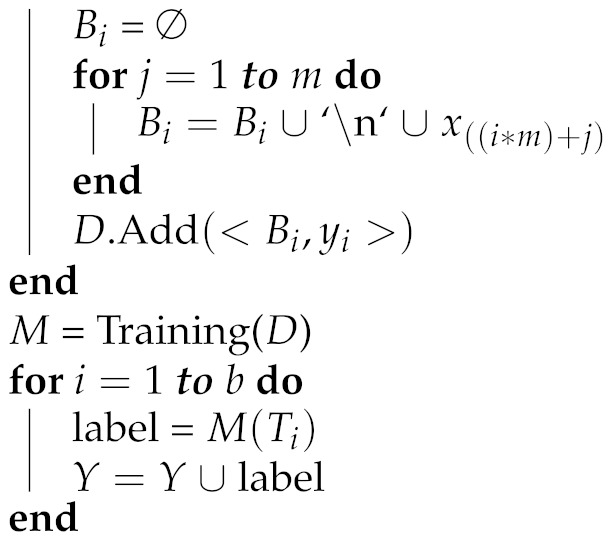


In this study, *entropy* was used to measure the homogeneity of the dataset when building a decision tree. At each node of the tree, the splitting criterion is the normalized information gain (difference in entropy). The entropy of a dataset is high if the number of samples in the classes is close to each other. On the other hand, the entropy value is small if there is a class that includes most of the samples in the dataset. The algorithm tries to minimize the entropy since a small entropy means that an instance can be classified with high probability. The entropy of a training data set *S* is calculated as follows:

(1) $$Entropy\left(S\right)=\sum_{i=1}^{k}P_{i}log_{2}P_{i}$$

where $P_{i}$ refers to the ratio of the samples belonging to class *i* and *k* is the number of the classes.

### 3.3. Advantages of the Proposed Approach

The proposed MOMIL approach has many advantages as follows:

- The proposed approach utilizes multi-instance learning to predict the match outcome in eSports for the first time. Thereby, it expands the standard classification task in the field of eSports.
- MOMIL is designed for team-level classification. This property increases the performance of the predictive model, since team collaboration is essential in completing game missions and achieving final success.
- eSports games are continuously updated, and the major game changes make the previous models obsolete. The proposed model overcomes this limitation by using data collected over short time periods.
- Another advantage of MOMIL is its implementation simplicity. After converting single-instance data into multi-instance data in a straightforward manner, any multi-instance learning algorithm can be applied easily.
- An important advantage of the proposed MOMIL method is that it is designed for analyzing any type of data that are suitable for win prediction. Therefore, it can be easily applied without having any background information. It does not require any specific knowledge or assumption for the given data. Therefore, it can be widely applied to many different eSports games such as LoL, Dota 2, Destiny, PUBG, and Counter-Strike.
- One of the key advantages is that it can be used for feature engineering which means identifying the most significant features for win prediction among the available features in the training bag set.
- Another advantage of MOMIL is its ability to deal with non-linear and complex win prediction problems.

### 3.4. Multi-Instance Classification Algorithms

In this study, the following nine multi-instance learning algorithms were tested and compared with each other.

**Multi-Instance AdaBoost (MIBoost)** [41]: As an upgraded version of the AdaBoost algorithm, it takes into consideration the geometric mean of the posterior of instances inside a bag. Naturally, the class label of a bag is predicted in two steps. In the first step, the algorithm finds instance-level class probabilities for the separate instances in a bag. In the second step, it combines the instance-level probability forecasts into bag-level probability for assigning a label to this bag.
**Multi-Instance Support Vector Machine (MISVM)** [42]: First, the algorithm assigns the class label of the bag to each instance in this bag as its initial class label. Afterward, it applies the SVM solution for all the instances in positive bags and then reassigns the class labels of instances based on the SVM result. The objective in MISVM can be written as follows:
  (2) $$\begin{array}{c} \min_{y_{i}}\min_{w,b,\varphi }\frac{1}{2}{\left\|w\right\|}^{2}+c\sum_{i}\varphi_{i}\\ s.t.\;\forall i:y_{i}\left(\left(w,x_{i}\right)+b\right)\geq 1-\varphi_{i},\varphi_{i}\geq 0,y_{i}\in \left\{-1,1\right\} \end{array}$$
  where *b* is the bias vector, *w* is the projection matrix, ${\left\{\varphi_{i}\right\}}_{i=1}^{N}$ are the slack variables of the support vector machine, and $y_{i}$ is the class label of the training pattern $x_{i}$.
**Multi-Instance Logistic Regression (MILR)** [43]: It performs collective multi-instance assumption by using logistic regression (LR) when evaluating bag-level probabilities. The instance-level class probability $S_{ij}$ with parameters θ=(w,b) is calculated as follows:
  (3) $$S_{ij}=\frac{1}{1+e^{-(w.B_{ij}+b)}}$$
  where *w* is a vector of feature weights, *b* is a bias parameter, $B_{ij}$ is the *j*th instance of the *i*th bag. The bag-level probability is estimated by using a *softmax* function, which combines the probabilities of the instances in the bag as follows:
  (4) $$S_{i}=p\left(C_{i}=1|B_{i}\right)=softmax_{\alpha }\left(S_{i1},\ldots ,S_{in}\right)=\frac{\sum_{j=1}^{n}S_{ij}e^{\alpha S_{ij}}}{\sum_{j=1}^{n}e^{\alpha S_{ij}}}$$
  where α is a constant related to the softmax approximation, $p(C_{ij}=1|B_{i})$ is the posterior probability that the *i*th bag is positive for binary classification, and *n* is the number of bags in the training set.
**Multi-Instance Tree Inducer (MITI)** [44]: This algorithm builds a decision tree in a best-first strategy by using a simple priority queue. It applies a splitting criterion to divide each internal node data point into subsets. At the beginning of the algorithm, a weight $w_{i}^{b}$ is assigned to each instance $x_{i}^{b}$ such that 1/|b| which is the inverse of the size of the bag *b*. After that, a weighted Gini-impurity metric is calculated for a set *S* as follows:
  (5) $$Gini\left(S\right)={\left(\sum_{x_{i}^{b}\in S}w_{i}^{b}\right)}^{2}-{\left(\sum_{x_{i}^{b}\in S^{-}}w_{i}^{b}\right)}^{2}-{\left(\sum_{x_{i}^{b}\in S^{+}}w_{i}^{b}\right)}^{2}$$
  where S− and S+ denote the negative and positive instances in *S*, respectively.
**Multi-Instance Rule Inducer (MIRI)** [45]: MIRI is an algorithm inspired by MITI. It is a multi-instance learner that benefits from partial MITI trees and generates a compact set of classification rules.
**Summary Statistics for Propositionalization (SimpleMI)** [46]: This method maps the instances in a bag to a single feature vector by analyzing statistical properties. Therefore, the basic idea is to transform a bag into a vector form first and then classify it by using a standard learner.
**Multi-Instance Wrapper (MIWrapper)** [47]: This algorithm assigns a weight to the instances in the bags. A class probability is calculated by the propositional model for each instance in the bag. After that, the predicted probabilities of instances are then averaged to assign a label to the bag.
**Citation K-Nearest Neighbours (CitationKNN)** [48]: It is an adapted version of KNN for MIL problems. To predict the class label of a query bag, the algorithm considers not only the nearest bags (called *references*), but also other bags that regard the query bag as one of their nearest neighbors (called *citers*). CitationKNN uses the Hausdorff measure to calculate the distance between two bags as follows:
  (6) $$Dist\left(B,B^{\prime }\right)=\min_{\begin{array}{c} 1\leq i\leq p\\ 1\leq j\leq q \end{array}}\left(Dist\left(b_{i},b_{j}^{\prime }\right)\right)=\min_{b\in B}\min_{b^{\prime }\in B^{\prime }}\left\|b-b^{\prime }\right\|$$
  where *B* and $B^{\prime }$ indicate two bags, i.e., $B=\{b_{1},b_{2},\ldots ,b_{p}\}$ and $B^{\prime }=\left\{b_{1}^{\prime },b_{2}^{\prime },\ldots b_{q}^{\prime }\right\}$; *p* and *q* are the number of instances in each bag, respectively; *b* and $b^{\prime }$ are two different feature vectors; and $b_{i}$ and $b_{j}^{\prime }$ are instances from each bag.
**Two-Level-Classification (TLC)** [49]: In the first level, the instances in each bag are re-represented by a meta-instance by encoding the relationships among them. In the second level, the algorithm induces a function to capture the interactions between the meta-instance and the class label of the bag. TLC requires the selection of a partition generator (i.e., C4.5) and a classifier (i.e., LogitBoost) with decision stumps [50].

## 4. Experimental Studies

To demonstrate the effectiveness of the proposed MOMIL approach, the experiments were carried out on a publicly available LoL dataset. The algorithms in the multi-instance learning package in WEKA [50] were used with default parameters. As a base classifier, the C4.5 Decision Tree (DT) algorithm was preferred due to its efficiency. This algorithm calculates entropy, which is a powerful measure to determine how a tree node splits data.

We used the 10-fold cross-validation technique to evaluate the performances of classifiers. The performances of the classifiers were compared in terms of accuracy and F-measure. Accuracy is a metric that is widely used in many applications [16,28,51] to measure the success of a model. Formally, *accuracy* is the proportion of correct predictions to total predictions. It is a useful measure of the degree of the predictive power of the classifier and how it may generally perform. In addition to accuracy, we also compared the performances of the methods in terms of F-measure. Although accuracy is a useful metric in classification performance, it alone is not sufficient to determine the quality of the prediction since it makes no distinction between the classes. The *F-measure* is a useful measurement since it takes into consideration three quantities: false-negative (FN), true-positive (TP), and false-positive (FP). F-measure is a summary performance measurement as it is the harmonic mean of precision and recall. Thereby, this metric represents both precision and recall by a single score.

Given the continuous changes and updates in eSports games, the strong connection between the features and target match outcome can remarkably limit model usage lifespan. For this reason, in this study, prediction models have evolved from season to season. Our approach, which builds the models on season-based data, is practically feasible since the models reflect game mechanics changes.

We conducted four experiments for demonstrating the efficiency of the proposed MOMIL approach.

*Experiment* 1—To show the superiority of MOMIL, we compared the multi-instance classification methods with their standard (single-instance) counterparts.
*Experiment* 2—We determined the best MIL algorithm by comparing alternative ones.
*Experiment* 3—We identified the most important factors that affect the victory in the matches in LoL.
*Experiment* 4—We compared our results with the results presented in the state-of-the-art studies [13,21,22,23,24] on the same dataset.

### 4.1. Dataset Description

To show the efficiency of MOMIL, the experiments were carried out on an LoL dataset publicly available in the Kaggle data repository (https://www.kaggle.com/paololol/league-of-legends-ranked-matches (accessed 21 November 2022 )). The dataset contains statistical match information of 1,834,520 players from the regions of North America and Europe. The raw data contain several tables with information about matches, players, champions, items purchased, team bans, and statistics. We combined the tables by using the join operation. After that, we extracted 40 features that captured relevant information to build a win-prediction model, including kills, assists, deaths, damages, rewards, and gold earned by each player. The objective was to predict the outcome of an LoL match based on team performances in the previous games.

In the data pre-processing stage, the complete data set was divided into subsets for season-based analysis (seasons 3, 4, 5, 6, and 7) since each season of the game has many changes that affect the winning strategy. The game is normally played with 10 players; for this reason, we removed the match records that contained fewer players. Although the raw dataset is not provided as multi-instance data, we transformed it for this purpose. The individual player statistics were taken into account as single-instance data, while team-level features were considered as multi-instance data. For winner prediction, the multi-instance method successfully maps input vectors representing game statistics of the players in the teams to output labels (win or loss). The winner can then be estimated for given query vectors of the players in a team.

### 4.2. Comparison of Single-Instance and Multi-Instance Classification

In the first experiment, to show the superiority of the proposed MOMIL approach, we compared the multi-instance learning methods with their standard (single-instance) counterparts, including MIBoost vs. AdaBoost, MISVM vs. SVM, MILR vs. LR, SimpleMI vs. DT, MIWrapper vs. DT, CitationKNN vs. KNN, and TLC vs. LogitBoost. It should be noted here that we compared both MIWrapper and SimpleMI with DT, because they apply a standard learner to multi-instance data, but in different ways, and in this study, we selected DT as the base learner for both of them. For this reason, we took into consideration MIWrapper vs. DT comparison, as well as SimpleMI vs. DT comparison.

Table 2 shows the results for each season separately in terms of accuracy (%). From the experimental results, it is clearly seen that multi-instance (MI) learning algorithms are better than their single-instance (SI) versions for all seasons. For example, MISVM (93.28%) achieved higher accuracy than SVM (88.99%) for season 3. On average, the multi-instance methods (MIBoost, MISVM, MILR, SimpleMI, MIWrapper, CitationKNN, and TLC) improved the classification accuracy by approximately 9.5%, 4.5%, 6%, 13.5%, 7.5%, 0.3%, and 11.3% compared to the single-instance methods (AdaBoost, SVM, LR, DT, KNN, and LogiBoost), respectively. Thereby, the results indicated that the proposed MOMIL approach could construct a robust model with high prediction accuracy. This is because it would make sense to see better performance in classification by applying multi-instance learning since cooperation among team members in LoL is crucial for success. In other words, MOMIL benefits from the collaboration property of multi-instance data and increases the performance of the predictive model.

One of the key properties of the MOMIL method is that it can be applied to any multi-instance dataset without having background information on the game. It is seen from Table 2 that the classification accuracy values of MI algorithms change from season to season. For example, MIBoost is more likely to correctly predict LoL match outcomes for season 7 (95.88%) than for season 4 (89.68%). Therefore, it experimentally confirmed that the performance of the multi-instance algorithm can be affected by the characteristics of seasons.

Although the MI algorithms have higher accuracy than their SI versions, the results were evaluated by using a statistical test to show that the differences in classification performances are statistically significant. The *p*-values for each season were calculated by using the Mann–Whitney-U test. According to the calculated *p*-values (0.0297, 0.0405, 0.0213) for seasons (3,5,7), 4, and 6, respectively, it can be concluded that the performance results are statistically significant because all the *p*-values are under the significance threshold level (α = 0.05).

**Figure 3:**
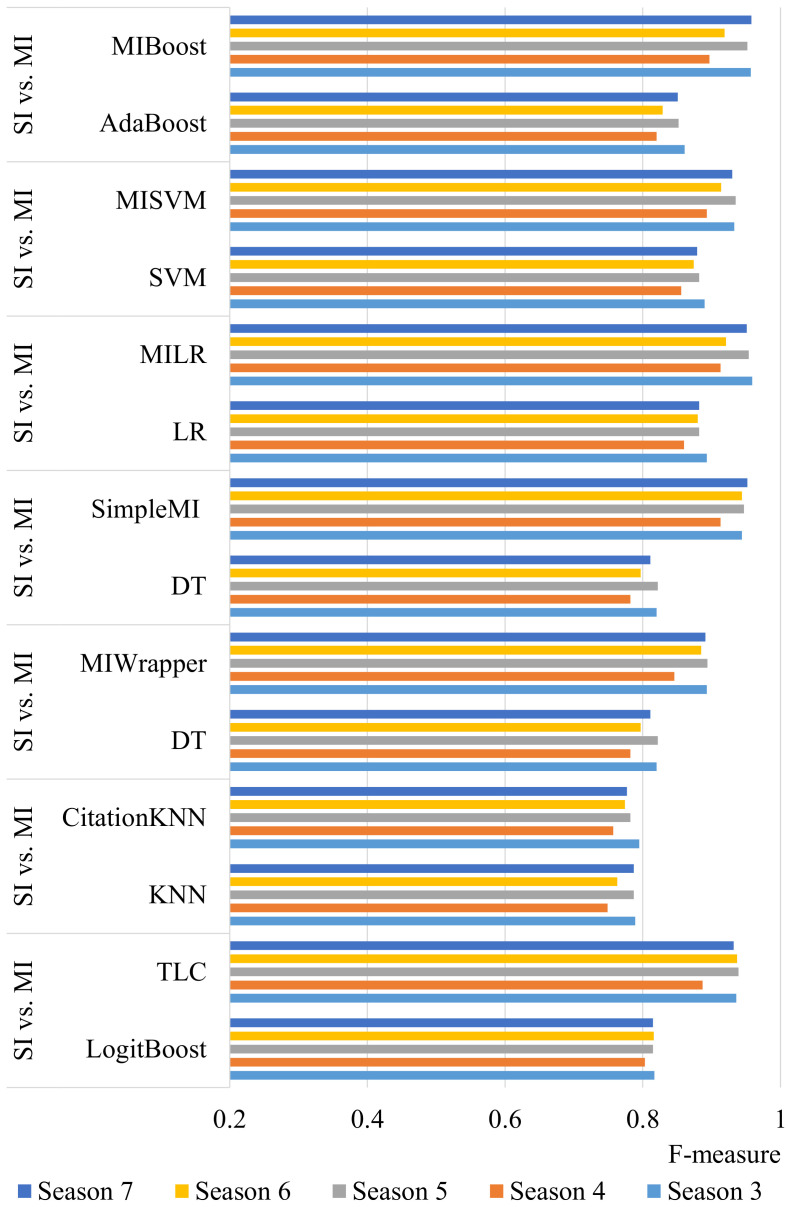
Comparison of single-instance (SI) and multi-instance (MI) classification methods in terms of F-measure.

Figure 3 shows the comparative results for MI and SI algorithms in terms of F-measure. According to the results, the MI methods achieved a higher F-measure value compared to the SI methods. The F-measure value ranges between 0 and 1, where 1 is the best value. In other words, the higher F-measure value, the better the classification performance.

As can be seen from Figure 3, the F-measure values obtained by the MI algorithms are closer to 1 than the SI algorithms for all seasons. The F-measure value difference between MI and SI is also remarkable for almost all algorithms, except CitationKNN. For instance, MILR (0.951) is significantly better than LR (0.882) for season 7. MI outperformed SI by increasing the F-measure by at least 0.008 points and at most 0.147 points on the seasons. One possible explanation for this improvement is that the MI methods analyze data at the team level, rather than only at the player level, since team cooperation is critical for match success. In other words, MI jointly considers the players in a team during the classification, since all five players are relevant for the team’s success and collaboration among players on each team significantly affects which team will win or lose. As a result, the proposed MOMIL approach can be successfully used for win prediction.

### 4.3. Comparison of Multi-Instance Classification Algorithms

**Figure 4:**
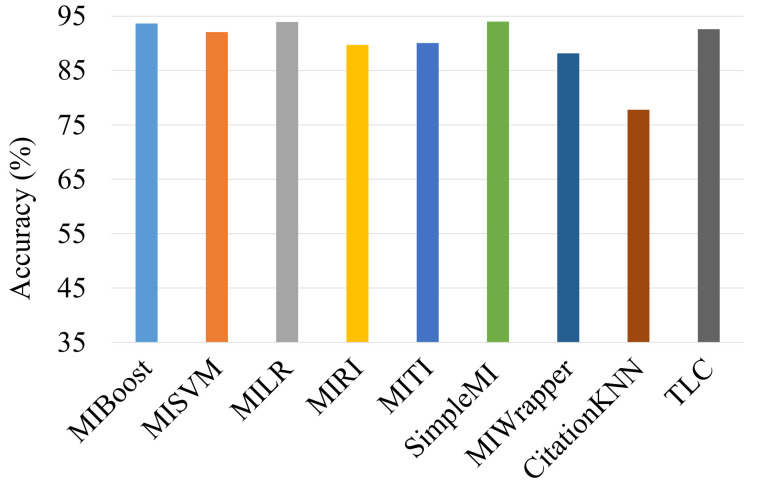
Comparison of multi-instance classification algorithms.

In the second experiment, we determined the best MIL method by comparing alternative ones. Figure 4 shows the average results in terms of accuracy (%). Based on the results, it can be noted that the SimpleMI method achieved the highest accuracy (94.01%) in terms of the seasonal average. The MILR and MIBoost methods followed it with accuracy values of 93.94% and 93.67%, respectively. However, the CitationKNN method performed poorly in comparison to the other methods. This is probably because of the fact that the closest references are likely similar to the closest citations and utilizing these did not improve prediction much.

### 4.4. Factor Analysis

**Figure 5:**
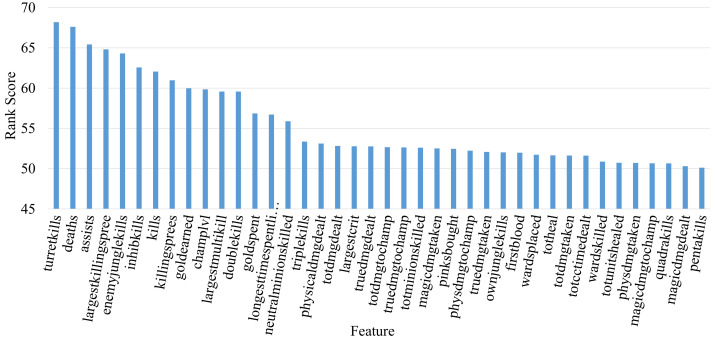
The importance of features for win prediction.

In the third experiment, we identified the most important factors that affect the victory of matches in LoL. Our aim is not only to build a well-performing win-prediction classifier but also to describe the prediction by means of feature importance. We used the OneR algorithms to evaluate the features since they rank features according to the minimum-error rate on the training set. Through pair-wise comparison, it estimates how each independent feature is correlated with the class attribute. In decreasing order, Figure 5 shows the importance of features for the win prediction in LoL matches. As can be seen, feature ranks range between 50.13 and 68.19. According to the results, the most important factors for win prediction are the number of turret kills, deaths per min, and assists per minute. The largest killing spree follows them as one of the important features. After killing-based features, gold earned by players is also ranked among the top 10 features.

### 4.5. Comparison of Our Study with the State-of-the-Art Studies

We compared our results with the results presented in the previous studies [13,21,22,23,24] on the same datasets. As shown in Table 3, the proposed approach outperforms the previous methods presented in [13,21,22,23,24]. For example, on average, MOMIL achieved significantly higher accuracy than the method in [13], which also focuses on win prediction.

In previous studies, deep learning techniques have also been applied to different eSports datasets. The results obtained in their studies can be summarized as follows. Lan et al. [39] proposed a CNN+RNN model for predicting win–loss outcomes and achieved 87.85% accuracy. Gu et al. [40] applied DNN to a different LoL dataset and obtained an accuracy of 62.09%. Similarly, Do et al. [52] also used DNN and reported that game outcomes can be predicted with 75.1% accuracy. Kim and Lee [53] proposed a deep learning model based on a bidirectional LSTM and reported 58.07% accuracy for win–loss prediction in LoL.

## 5. Conclusions and Future Work

The objective of this study was to successfully predict the outcome of an eSports match by using machine learning methods. For this purpose, we proposed a new approach, called MOMIL, which considers the task of analyzing data not only to handle the win prediction probability of individual players but also to explore the winning probability of a team as a whole. In particular, each player is related to all the teammates; therefore, our approach makes a team-level analysis, where the feature vectors of the players in a team are considered together.

In the experiments, our approach was applied to a publicly available LoL dataset that has a variety of input features that represent different respects of matches, including kills, assists, deaths, damages, rewards, and gold earned by each player. We built the models on season-based data since each season of the game has many changes that affect the win-prediction model.

The main findings from this research can be summarized as follows:

- The experimental results showed that the multi-instance-based classification approach outperformed the standard classification approach for winner prediction in terms of accuracy and F-measure.
- The proposed MOMIL approach achieved up to 95% accuracy for match outcome prediction in LoL seasons.
- Among multi-instance learning algorithms, the SimpleMI method achieved the highest accuracy in terms of the seasonal average. The MILR and MIBoost methods followed it.
- The most important factors for win prediction are the number of turret kills, deaths per min, and assists per minute.

Our study especially investigated the winning predictors in League of Legends. However, as future work, it can also be applied to similar MOBA games such as Dota 2 and Counter-Strike.

## Figures and Tables

**Table 1:** Comparison of our study with the previous studies.

Ref.	Year	Description	Task			Algorithm	Game			MIL	Season
			CF	R	CL		LoL	Dota2	Other		Based
[26]	2023	Predicting match outcome	√			SVM, NB, KNN, NN	√			X	X
[25]	2022	Predicting match outcome	√			LSTM		√		X	X
[8]	2021	Predicting whether the player will win or lose	√			LR, KNN, NN	√			X	X
[9]	2021	Predicting in-game win probability	√			LR	√			X	X
[27]	2021	Predicting the placement of players		√		DT, LR, SVM			PUBG	X	X
[28]	2021	Classifying eSports games	√			DT, NB, RF	√	√	CS	X	X
[14]	2020	Predicting match outcome		√		BHR		√		X	X
[11]	2020	Modelling the results (win/lose) of the game		√		NN	√			X	X
[16]	2020	Predicting the player skill and re-identification	√			LR, KNN, SVM, RF, NB	√			X	X
[12]	2020	Predicting match outcome	√			CNN, NN, CART	√			X	X
[18]	2020	Predicting the players’ rankings		√		RF, GBM			PUBG	X	X
[10]	2020	Predicting match outcome	√			GBM, RF, LR		√		X	X
[23]	2020	Recommending items	√			DT, LR, NN, CNN	√			X	X
[13]	2019	Predicting match outcome	√			KNN	√			X	X
[29]	2019	Identifying roles			√	K-Means		√		X	X
[17]	2019	Predicting the player skill	√			ERT			CS	X	X
[7]	2019	Predicting match outcome	√			CART, LR		√		X	X
[6]	2019	Predicting match outcome	√			LR, RF, GBM		√		X	X
[19]	2019	Predicting a player will die in the next 5 s	√			DNN		√		X	X
[24]	2019	Recommending items	√			Apriori, Eclat, DT, LR, NN	√			X	X
[30]	2018	Clustering player-centric networks			√	Affinity Propagation	√			X	X
[31]	2018	Predicting round results	√			DT, RF, KNN, PCA			CS	X	X
[32]	2018	Predicting player churn		√		MECR	√			X	X
[15]	2017	Predicting match outcome	√			LDA, QDA, SVM, KNN, Weighted KNN			Star Craft	X	X
Proposed		Predicting match outcome	√			MIBoost, MISVM, MILR, MIRI, MITI, SimpleMI, MIWrapper, CitationKNN, TLC	√			√	√

**Table 2:** Comparison of single-instance (SI) and multi-instance (MI) classification methods for each season in terms of accuracy (%).

	MIBoost	AdaBoost	MISVM	SVM	MILR	LR	SimpleMI	DT
Season 3	**95.69**	86.09	**93.28**	88.99	**95.88**	89.32	**94.45**	81.98
Season 4	**89.68**	81.98	**89.28**	85.58	**91.29**	86.03	**91.29**	78.18
Season 5	**95.23**	85.16	**93.45**	88.22	**95.39**	88.19	**94.74**	82.16
Season 6	**91.87**	82.87	**91.37**	87.41	**92.07**	88.01	**94.38**	79.74
Season 7	**95.88**	85.06	**93.03**	87.92	**95.06**	88.17	**95.21**	81.14
	**MIWrapper**	**DT**	**Citation** **KNN**	**KNN**	**TLC**	**Logit** **Boost**		
Season 3	**89.26**	81.98	**79.53**	78.92	**93.63**	81.75		
Season 4	**84.58**	78.18	**75.87**	74.91	**88.74**	80.27		
Season 5	**89.43**	82.16	78.27	**78.71**	**93.93**	81.50		
Season 6	**88.55**	79.74	**77.51**	76.37	**93.67**	81.61		
Season 7	**89.06**	81.14	77.76	**78.70**	**93.16**	81.49		

**Table 3:** Comparison of our study with state-of-the-art studies.

Ref.	Year	Algorithm	Existing Method	Proposed Method
			F-Measure	F-Measure
[21]	2022	Multi-attribute Context-aware Item Rec. (MCIR)	0.616	0.903
			F-Measure	F-Measure
[22]	2022	Relation-aware Graph Attention Network	0.626	0.903
			F-Measure	F-Measure
[23]	2020	Decision Tree Logistic Regression Artificial Neural Network Convolutional Neural Networks Team-aware Transformer-based Item Rec. (TTIR)	0.379 0.468 0.566 0.586 0.596	0.903
			F-Measure	F-Measure
[24]	2019	Apriori Equivalence Class Transform. Decision Tree Logistic Regression Artificial Neural Network	0.48 0.48 0.38 0.43 0.53	0.903
			Accuracy	Accuracy
[13]	2019	K-Nearest Neighbors	58.00%	90.23%

## Data Availability

The LoL dataset is publicly available at the following website: https://www.kaggle.com/datasets/paololol/league-of-legends-ranked-matches (accessed on 20 November 2022).
